# COVID-19 Vaccination and Late-Onset Myasthenia Gravis: A New Case Report and Review of the Literature

**DOI:** 10.3390/ijerph20010467

**Published:** 2022-12-27

**Authors:** Eleonora Virgilio, Giacomo Tondo, Claudia Montabone, Cristoforo Comi

**Affiliations:** 1 Department of Translational Medicine, Section of Neurology, University of Eastern Piedmont, 28100 Novara, Italy; 2 Neurology Unit, Department of Translational Medicine, S. Andrea Hospital, University of Piemonte Orientale, 13100 Vercelli, Italy

**Keywords:** adverse drug events, SARS-CoV-2, vaccine, neuromuscular junction, Ach receptor, autoimmune diseases, vaccination hesitancy

## Abstract

Myasthenia gravis (MG) is a rare autoimmune disease that is potentially threatening for patient life. Auto-antibodies targeting structures of the neuromuscular junction, particularly the acetylcholine receptor (AchR), are often found in the serum of MG patients. New-onset MG after SARS-CoV-2 vaccination has rarely been reported since the introduction of vaccination. Infections and COVID-19 infection have also been reported as possible triggers for a myasthenic crisis. We report a case of new-onset MG after receiving the mRNA COVID-19 vaccination. The patient was a 73-year-old male initially presenting with ocular symptoms and a rapid generalization. We also performed a literature revision of 26 described cases of MG after SARS-CoV-2 immunization. The patients were a majority of males with generalized late-onset MG occurring after the first dose of vaccine, similar to our patient. Only our patient showed a thymoma. Thymic mass and the positivity of AchR antibodies suggest that vaccination might have triggered a subclinical pre-existing MG with symptoms flaring. Clinicians should be aware of possible new-onset MG after COVID-19 vaccination, particularly in at-risk patients. Even though COVID-19 vaccination should be recommended in MG patients, particularly in well-compensated patients. However, more studies need to be performed in the future.

## 1. Introduction

Since March 2020, after the World Health Organization (WHO) declared a global pandemic caused by the novel coronavirus disease 2019 (COVID-19), an enormous number of people have been infected, resulting in morbidity and mortality [1]. The pandemic is still considerably impacting health care and society in general. Thankfully, the introduction of a vaccination against SARS-CoV-2 has reduced the spread of the virus and death rates [2]. However, since the global vaccination program began, much interest has also been raised about the short- and long-term effects of COVID-19 vaccination [2]. Even though their safety has been proven to be satisfactory in randomized clinical trials, [3], in 2022, severe and unexpected neurological complications were reported: Guillain Barré syndrome, cerebrovascular events, and autoimmune diseases including myasthenia gravis (MG) [2]. Several vaccines are now available such as the Pfizer-BioNTech (Cominarty/BNT162b2) and Moderna (mRNA-1273) vaccines from December 2020, the Oxford-AstraZeneca ChAdOx1 (AZD1222, Vaxzevria) vaccine, and many more. The evidence of possible adverse events related to COVID-19 vaccination has further boosted vaccine hesitancy or refusal despite availability [4]. Specific criteria to determine the causality assessment of an adverse event following immunization (AEFI) are available from the WHO, which allows for their categorization as certain, probable, possible, unlikely, conditional/unclassified, and unassessable/unclassifiable relationships [5,6]. The system relies on four steps. Even though these assessments cannot produce a precise and reliable quantitative estimation of relationship likelihood, a causality assessment has become a common routine procedure in pharmacovigilance, but is rarely used by clinicians [5,6].

MG is a rare autoimmune disorder of the neuromuscular junction caused, in most cases, by antibodies to the acetylcholine receptor (AchR) [7]. More rarely, antibodies against muscle-specific kinase (MuSK-Ab) or lipoprotein-related protein 4 (LRP4) can be detected in the serum of the patients. The absence of all three (AchR, MuSK, and LRP4) antibodies defines a “triple seronegative” patient [8]. Antibody detection is fundamental to confirming MG diagnosis and follow-up [9]. Many laboratory tests are available such as the enzyme-linked immunosorbent assay (ELISA), cell-based assays (CBA), or radioimmunoassay (RIA) [10]. RIA is, at the moment, the gold standard method, performed with “homemade” methods or commercial kits with extremely high specificity, nearly 100% for both AChR and MuSK. RIAs are also highly sensitive but require radioactivity [10]. ELISAs are often used as an alternative due to being easier to perform and not requiring radioactivity [11]. However, their performances, particularly sensitivity, seem to be inferior compared to RIA. Recently, live, and fixed CBA can detect additional low-affinity antibodies to clustered AChR, MuSK, and LRP4 [12,13]. However, positivity rates are variable, and both the clinical relevance and utility of CBA platforms remain unclear, even though several studies suggest that CBA may be useful in the serologic evaluation of RIA-negative samples due to higher sensitivity. It was proposed that some patients may have autoantibodies that recognize epitopes exposed when AchR is aggregated on the cell surface, therefore, identified with CBAs [9,10,12,13]. The diagnosis of seronegative MG can be challenging, since many conditions may mimic MG. Consequently, an adequate clinical and electrophysiological evaluation is mandatory to support the diagnosis. The classical manifestation of MG is a fatigable weakness affecting striated muscles, resulting in signs and symptoms fluctuating within the same day, but also from day to day and even within longer time-frames [7]. Symptom presentation can vary, ranging from an ocular onset to oculobulbar, bulbar, or generalized disease. Infections, stress, drugs, and vaccines can trigger this condition, presenting life-threatening symptoms requiring hospitalization. Patients with MuSK antibodies have more frequent bulbar symptoms, whereas LRP4 normally displays a milder disease course [7,10]. Finally, most seronegative patients have a mild disorder with predominant ocular manifestations [10]. MG can present either in young women or elderly patients. Late-onset MG (LOMG) is more frequent in males than adult-onset MG, with a peak at 70 years old [14]. There is no specific definition for LOMG patients: some studies consider LOMG patients with onset above 50 years old [15,16] and others above 60 years old [14,17,18]. Thymic dysfunction is a well-recognized co-factor of the disease, and MG patients may have a family history of autoimmune illnesses. Thymoma, but no other thymic tumors, is particularly associated with MG. Thymic hyperplasia is reported in most patients with early-onset MG and in some patients with late-onset, ocular MG, and seronegative disease [15,16]. Here, we present a case of late-onset MG with thymoma after the first dose of the AstraZeneca COVID-19 vaccine and a literature review of all reported cases from December 2020 to October 2022.

## 2. Materials and Methods

### 2.1. Literature Revision

**Figure 1:**
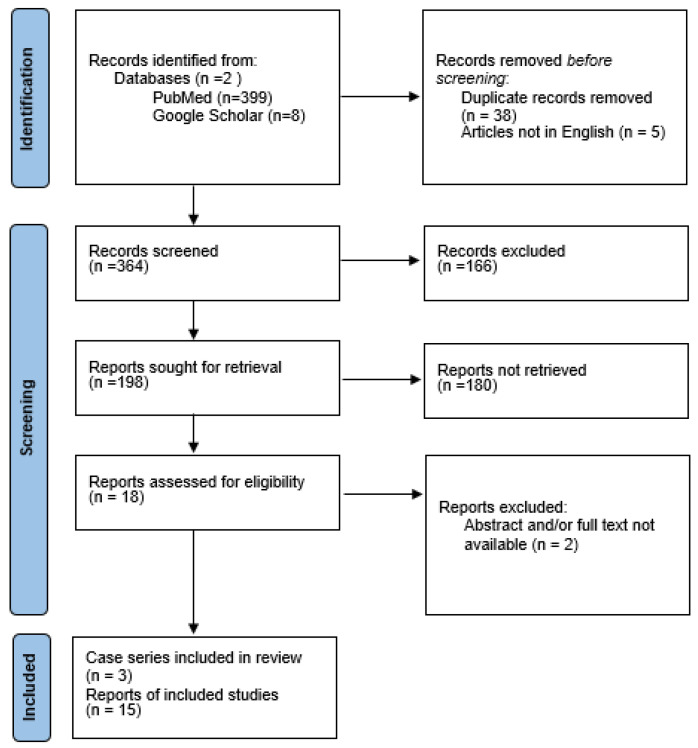
Flowchart of the literature research.

We performed a literature review through a search of the PubMed and Google Scholar library databases through several MeSH terms: [vaccination] and [myasthenia gravis], [vaccination] and [MG] [vaccines] and [myasthenia gravis]; [vaccines] and [MG] and [COVID-19] or [Sars-Cov-2]. Studies or reports were included if published before 31 October 2022, with an English language restriction. We considered eligible cases describing patients with a new-onset MG following SARS-CoV-2 vaccination with no restriction of time from immunization. We excluded patients with pre-existing MG or with new-onset MG after SARS-CoV-2 infection (Figure 1).

### 2.2. Statistical Analysis

All statistical analyses were performed using GraphPad Prism 9 for Windows (GraphPad Software, La Jolla, CA, USA). We presented the continuous data as the mean and standard deviation (SD), categorical data as the median and range, and proportions as numbers with the corresponding percentage. The data distribution was preliminarily assessed, and resampling methods were not conducted. Unpaired t-test with Welch’s test, Mann–Whitney U test, and Kruskal–Wallis test were used to compare the continuous variables; the chi-squared test and Fisher test were used for the categorical variables. No particular post-hoc analysis was added. 

## 3. Results

A total of 196 possible results were found. Two independent authors (E.V. and G.T.) screened the titles and abstracts. After removing redundant titles, articles not pertinent, and articles not in English, a final selection of 18 papers was included. Therefore, in addition to our case, we collected 28 patients with post-vaccinal MG until October 2022. Two of the 18 papers (corresponding to two patients) were later excluded for lack of the full-text and abstract. The results of our literature review are reported in Table 1.

### 3.1. Case Presentation

**Figure 2:**
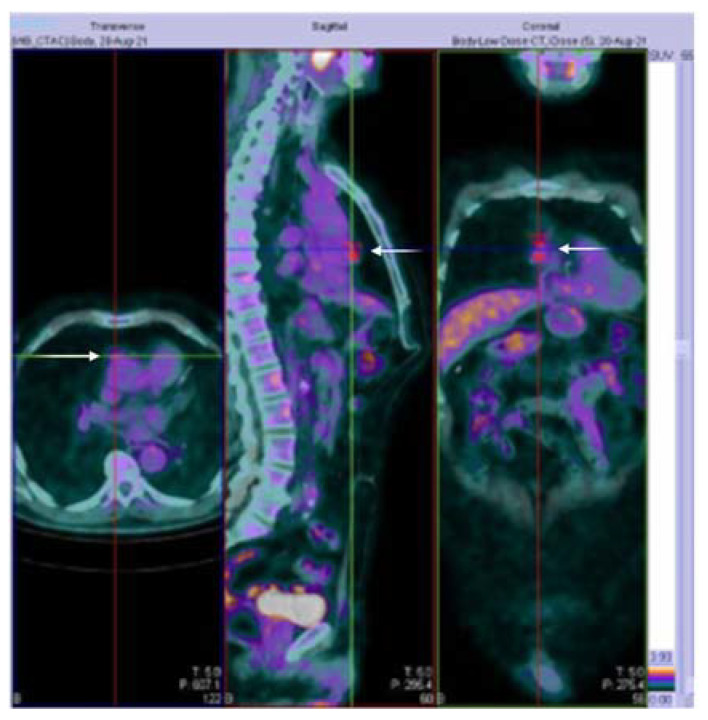
Positron emission tomography (PET)-CT of our patient. Fused 18fluorodeoxyglucose [18F]FDG-PET and CT transverse, sagittal, and frontal images confirming a moderate [18F]FDG uptake in the anterior mediastinum (white arrows) with a maximum standardized uptake value (SUV) of 3.16, consistent with the suspicion of ectopic mass (most probably thymoma). The SUV is the ratio of the image-derived radioactivity concentration and the whole-body concentration of the injected radioactivity, and some authors have suggested that SUV is increasingly higher in thymic mass according to the WHO classification [19]. The histologic analysis confirmed the thymoma diagnosis.

A 73-year-old man with a history of hypertension under stable treatment (low dose ramipril 2.5 mg/day) and type II diabetes (treated only with diet) reported several intermittent episodes of ptosis in the left eye associated with diplopia. Four weeks before, he was administrated with the first dose of the Oxford-AstraZeneca ChAdOx1 vaccine. He denied any symptoms of muscular fatigue prior to the injection. His symptoms occurred mostly in the evenings watching television. In the following months, he executed a cranial MRI without any pathological alteration, with his blood exams showing an elevated glycated hemoglobin of 56 mmol/mol (normal values ranging between 20 and 42 mmol/mol) and a fasting glucose of 155 mg/dL as well as a neurological evaluation. Considering the fluctuation of the symptoms, we proposed an electrophysiological examination, a Hess–Lancaster test, and the AchR antibodies serum research (dosed with ELISA) with the suspicion of a neuromuscular junction disease. He later clinically worsened with bulbar and superior limb weaknesses and was admitted to our neurology department three months after the injection. Repetitive stimulation in the facial and anconeus muscles confirmed the generalized MG clinical diagnosis. Thoracic CT demonstrated a thymic mass without infiltration of surrounding structures, later established by a PET-CT (Figure 2). Serum AchR antibodies tested positive.

During their hospitalization, treatment with pyridostigmine orally, initially at 120 mg/daily, then increased to 360 mg, was started with rapid clinical improvement. There was no need for immunoglobulin iv, and oral steroids were introduced at a lower dosage of 12.5 mg, considering the weight of 70 kg of the patient and the history of type II diabetes. At discharge after 16 days, examination of the patient revealed only mild bilateral ptosis of 20% after muscle activation. The patient was then started with oral azathioprine, 75 mg per day. Later, the thymic mass was removed, confirming the thymoma diagnosis (AB type according to the WHO classification, or mixed type according to the Müller–Hermelink classification and pT1a TNM staging) [20]. Eight months after the diagnosis of MG, he was scheduled for Moderna vaccination without side effects. One year after diagnosis, we observed a complete remission of neurological symptoms with oral azathioprine 100 mg and piridostimine 225 mg, if we apply the WHO causality assessment checklist.

### 3.2. All 26 Reported Patients

**Figure 3:**
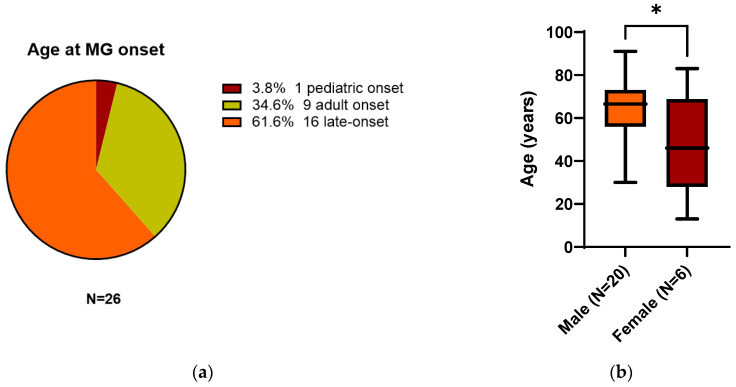

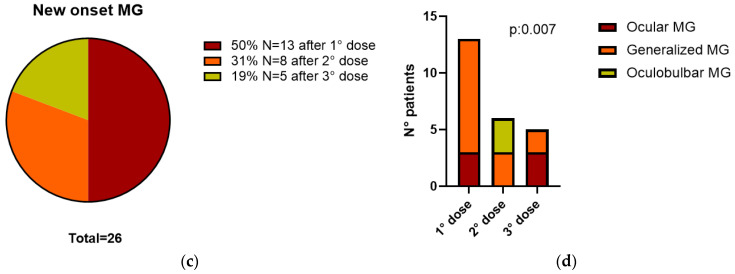
(**a**) Distribution of patients according to the age at MG onset and diagnosis. (**b**) In the literature, the majority of MG new diagnoses after COVID-19 vaccination occurred in males with higher age (* *p* < 0.05) at onset and diagnosis (LOMG). (**c**) Distribution of patients according to the dose of vaccine and onset of MG. (**d**) Stratification of patients based on the dose of immunization and type of MG. Resampling methods were not conducted. Abbreviations: MG: myasthenia gravis, LOMG: late-onset MG.

Patients presented a mean age of 60.8 years old (±SD 18.6 years), but interestingly, 16/26 (61.6%) could be classified as late-onset MG (LOMG) defined as an onset ≥60 years old, whereas 9/26 (34.6%) showed adult-onset (between 18 and 59 years old), and only one patient (3.8%) displayed a pediatric onset at 13 years old (Figure 3a). When considering sex, we found a higher prevalence of males than females. Twenty patients out of 26 (77%) were male, with a mean age of 64.8 years old (±SD 15 years). Only 6/26 (23%) were female and were significantly younger than males (mean age 47.5 ± SD 24.2 years, p:0.04, Figure 3b). Six patients (23%) were classified as ocular MG, three patients (11.5%) as oculobulbar, 15 patients (58%) as generalized, and two (7.5%) as not reported. Only our patient (3.8%) was diagnosed with thymoma and two adult females (7.7%) with thymic hyperplasia. However, results of a CT thoracic scan were missing in 10 patients (38.5%). Generally, the patients improved over time with specific treatments, but data were highly missing, especially on the long-term follow-up (unlikely our patient), even though two patients (7.7%) were intubated. Thirteen patients (50%) had symptom onset following the first dose, eight (31%) after the second dose, and five (19%) after the third (booster) dose of the vaccine (Figure 3c). Nineteen (73%) patients were administered mRNA vaccines (15 with Pfizer-BioNTech and four with Moderna mRNA-1273), six (23%) with Oxford-AstraZeneca ChAdOx1, and only one (4%) with Sinopharm. The mean time from SARS-CoV-2 vaccination to MG symptom onset was seven days (±6 days with a range from 0 to 28 days). The majority of patients, 16/26 (61.5%), developed symptoms within the first week of vaccination, seven patients (27%) in the second week, and only three patients (11.5%) ≥14 days from immunization. No statistically different days of latency to onset were observed when differentiating the age or sex of the patients. When stratifying patients based on the dose of immunization, we found no difference in the age of the patients (even though patients with MG after the first dose showed a tendency of younger age at onset p:0.06) and days between immunization and neurological manifestations. On the other hand, generalized MG was significantly more frequent after the first dose (chi-square 13.9 df4 p:0.007, no post-hoc analysis performed; Figure 3d). Reports included patients in the USA, Europe, and Asia. Finally, most diagnosed patients displayed AchR+ antibodies (21/26 81%). No cases of antibodies against MuSK or LRP4 were found in the literature. Only three (11%) were AchR negative (one tested only for AchR, one also for MuSK–double-seronegative, and only one triple-seronegative). For two patients (8%), the data were not specified. The type of diagnostic assay used was rarely specified.

## 4. Discussion

MG is a rare neuromuscular disease. Diagnosis of MG AchR positive (which corresponded to almost 80% of the cases) is usually based on clinical features, electrophysiological exams to confirm defects in neuromuscular transmission, and positivity in serological tests. The complete pathophysiology of the disease is yet to be elucidated. Here, we report the first patient with new LOMG associated with thymoma within four weeks from SARS-CoV-2 vaccination. Our patient also suffered from well-controlled hypertension treated with a stable low-dose ace-inhibitor and mild type II diabetes, which have not been reported in the literature to be associated with MG, or a higher rate of COVID-19 vaccination neurological autoimmune side effects. Moreover, we also discussed 26 cases reported in the literature up until October 2022. Our findings indicate that older males may be at risk of a generalized LOMG following COVID-19 vaccination (particularly within the first week after the first dose). Usually, MG is more common in young females, and LOMG normally represents one-third of all MG patients, with a higher rate in males [7,14,16]. Conversely, we observed a higher prevalence of male LOMS compared to young adult-onset females. LOMG is defined according to different classifications as onset after 50 years old or after 60 [14,15,16,18]. We applied the cutoff of 60 years old, but if patients above 50 years old are considered, only 23% of reported new-onset MG post-immunization would have been adult or juvenile. Clinically ocular MG is less frequent than generalized (usually reported in 15% of patients); 80% of MG patients are usually AchR positive, and 10–15% present thymoma. We confirmed all of these clinical characteristics except for thymic pathology, which was less represented (only our patient was diagnosed with thymoma). 

Globally, MG is a relatively uncommon disorder with an annual incidence of approximately seven to 23 new cases per million [21]. The prevalence is about 70 to 320 per million [21], but has been increasing in the last decades due to better diagnosis, population aging, and the longer life span of affected patients [22]. Infections with the risk of myasthenic crisis may aggravate MG. A myasthenic crisis is a life-threatening exacerbation of MG, defined as the worsening of muscular weakness requiring intubation or noninvasive ventilation [23]. Similarly to other infections, COVID-19 infection may also trigger a myasthenic flare [24]. Cases of MG following vaccinations have been rarely reported in the literature, and only a few reports of MG following human papillomavirus or hepatitis B vaccinations have previously been published [25,26]. Therefore, considering the worldwide immunization rate [27], we can assume that a new onset of MG post-COVID-19 vaccination is a rare adverse event [2]. Unfortunately, considering the high rate of missing data in the analyzed cases, application of the WHO causality assessment checklist was impossible. Conflicting results exist in the literature regarding the possibility that previously diagnosed MG patients may be aggravated following vaccination against COVID-19 [28,29,30]. In Japan, Ishizuchi et al. observed a disease flare preferentially in younger MG patients in a large cohort of patients with treatment escalation induced by COVID-19 vaccines in 1.0% of the total cohort [28]. The authors concluded that patients with severe bulbar symptoms and a history of myasthenic crisis should postpone COVID-19 vaccination [28]. A Chinese survey-based study found SARS-CoV-2 vaccines to be safe in stable MG patients. Worsening after vaccination was more frequently seen in patients with intervals since the last aggravation of ≤6 months [29]. This was also confirmed by a systematic review including papers from January 2000 to October 2022 highlighting that COVID-19 infection could increase the risk of new-onset MG, myasthenic crisis, respiratory failure, and mortality rate, probably due to cytokine storm [24]. However, a review by Sansone et al. concluded that COVID-19 vaccination is safe overall in MG and the benefits outweigh the risks, even if the exacerbation of MG symptoms with variable severity could be present in up to 8–9% of the cases [30]. Therefore, MG symptoms and signs of transient worsening or disease fluctuation should be carefully monitored, especially post-vaccination. In contrast, a retrospective study conducted in Israel concluded that COVID-19 is hazardous for generalized MG patients, with a higher risk than the general population of disease worsening and mortality, especially during the Alpha and Delta waves [31]. Vaccination did not raise the risk for exacerbation and was associated with a reduced risk for severe COVID-19 [31]. These conclusions also seem to have been confirmed by other studies on smaller sample sizes [30,31,32,33,34]. Patone et al., in a large population-based study of more than 32 million people, observed an increased risk of hospital admission in MG who received the Oxford-AstraZeneca ChAdOx1 vaccine in a window of 15–21 days. However, no association was identified with the Pfizer-BioNTech vaccine [35]. In our literature review, we found both cases following mRNA vaccination and non-mRNA immunization. The prognosis was overall good, and most patients displayed a good outcome after MG diagnosis and the start of treatment. 

The pathological mechanism underlying this possible association is still debated: a possible bystander activation has been hypothesized [36]. Vaccination may cause the release of previously sequestered self-antigens, resulting in the activation of autoreactive T-cells [36]. Alternatively, molecular mimicry mechanisms may be involved [36]. SARS-CoV-2 proteins in vaccines might cross-react with AchR target proteins, causing a clonal activation of B lymphocytes and therefore MG. Genetic predisposition or susceptibility is probably necessary to initiate this mechanism. Some of the reported cases probably already had the disease at a presymptomatic stage, and immunization only served as a co-factor. The production of AChR antibodies depends on T cells stimulating B cells to produce autoantibodies, which probably occur in an intrathymic environment [7,16,21]. Therefore the antibodies’ positivity against AchR a few days after immunization and the presence of thymic mass suggest that vaccination aggravated and triggered the autoimmune disease in at-risk or undiagnosed patients. Finally, autoimmune/inflammatory syndrome induced by adjuvants (ASIA) has been proposed as a possible pathogenetic mechanism in other autoimmune diseases such as thyroiditis [37]. Adjuvants enhance the immunogenicity of vaccines and increase both innate and adaptive immune responses. However, no evidence is present of ASIA and MG. One possible proof of the causal relationship between MG and COVID vaccination would be the availability of negative serum samples of the patients before immunization in a prospective study setting. To date, we have only found one small case series including seven patients with new onset MG following COVID-19 immunization [38], or otherwise only single case reports. Only one systematic review can be found in literature that considered four articles (five patients) from inception to 26 March 2022 [39]. The authors could not conclude whether MG is necessarily a complication of the different COVID-19 vaccines [39]. 

Our article presents several limitations: first of all, the limited sample size of the reported patients indicates that the results may be not representative of the population, and second, all of the heterogeneity concerning the administered vaccines may have influenced our results. The large amount of missing data both regarding the clinical presentation, the diagnosis, and the follow-up of the patients is also an issue and limited the possibility of using standardized causality checklists. Finally, the statistical analysis used without bootstrapping techniques also needs to be considered. Therefore, more data must be collected to draw solid conclusions and define a causal relationship. Registry-based studies may provide the large sample sizes that are needed, however, these type of studies often lack specific information regarding the disease (i.e., clinical manifestation, tests used to achieve diagnosis, long follow-up). Prospective studies to study a possible causal relationship between MG and COVID vaccination would need the availability of negative serum samples of the patients before immunization in a large prospective study setting, which is highly difficult to achieve. Moreover, considering that concerns about the neurological side effects of vaccines is an old discussion, we can learn from previous experiences with other vaccines in MG and other immune mediated diseases. Another approach should focus on the implementation of studies to compare the effects on the disease and general population of COVID-19 vaccines and COVID-19 infection itself as well as compare between different types of immunization.

## 5. Conclusions

Mass vaccination represents the turning point against the pandemic. However, adverse reactions such as the possibility of autoimmune disease triggered by vaccination might lead to reduced vaccine acceptance and hesitancy. Data in the literature are still lacking for rare diseases such as MG, and at the moment, only a small number of cases of Mg following COVID-19 immunization have been reported. Therefore, solid conclusions cannot be formulated. However, given the possible risks of COVID-19 infection on already diagnosed patients, the benefits of vaccinations could possibly outweigh the risks. Nonetheless, clinicians should be aware that the presence of fatigability after SARS-CoV-2 immunization raises the suspicion of a MG diagnosis.

**Table 1:** Characteristics of the new-onset cases with myasthenia gravis (MG) cases in timely association with SARS-CoV-2 vaccination.

No.	Age/Sex	Vaccine Name/Dose	Days Onset	Abs Status	First Symptom	MG Type	Treatment	Follow-Up	Chest-CT	Ref.
1	65/M	Pfizer-BioNTech/3°	21	AchR+	Diplopia	Ocular	P300 mg/S10 mg	Good recovery at 2MTHs	no	[40]
2	82/M	Pfizer-BioNTech/2°	2	AchR+	Slurred speech	Generalized	IV-P/IV-IG/S	Improved	no	[14]
3	91/M	Pfizer-BioNTech/2°	10	AchR+	Oculobulbar	Oculobulbar	P90 mg	Unchanged	no	[41]
4	80/M	Moderna mRNA1273/2°	6	AchR+	Oculobulbar	Oculobulbar	P90 mg/PLAEX/AZA150 mg	Mild ptosis at 3MTHs	no	[41]
5	55/M	Moderna mRNA1273/1°	3	AchR+	ULs-neck-diplopia	Generalized	P240 mg/IV-IG/S50 mg	Mild UL at 3MTHs	no	[41]
6	73/M	O.A ChAdOx1/1°	8	AchR and RF+	Monolateral ptosis	Ocular	P240 mg	NR	no	[42]
7	30/M	Moderna mRNA1273/1°	2	AchR+	Diplopia	Generalized	P90 mg/S10 mg	NR	no	[26]
8	35/M	O.A ChAdOx1/1°	7	AchR+	Diplopia	Ocular	NR	NR	no	[43]
9	33/F	Pfizer-BioNTech/2°	0	Double-seronegative	GW and diplopia	Generalized	P360 mg	Partial improvement	TH	[44]
10	72/M	Pfizer-BioNTech/2°	1	NR	NR	NR	S60 mg/PLAEX	Recovered	NR	[45]
11	73/M	Pfizer-BioNTech/2°	7	NR	Ocular signs	Generalized	PLAEX/P/S	NR	NR	[45]
12	65/M	Pfizer-BioNTech/3°	3	AchR+	Diplopia	Ocular	PLAEX/P180 mg	Improvement at 3MTHs	no	[46]
13	60/M	Moderna mRNA-1273/3°	6	AchR and ANA+	Dysarthria	Generalized	P/S	Improvement	no	[47]
14	13/F	Pfizer-BioNTech/1°	14	AchR negative	NR	Generalized	P/S	NR	NR	[38]
15	59/M	O.A ChAdOx1/1°	2	AchR+	NR	Generalized	P/S	NR	NR	[38]
16	63/M	Pfizer-BioNTech/3°	3	AchR+	NR	Ocular	P	NR	NR	[38]
17	73/M	Pfizer-BioNTech/3°	12	AchR+	NR	Generalized	P/IV-IG/S	NR	NR	[38]
18	50/M	Pfizer-BioNTech/1°	7	AchR+	NR	Ocular	P	NR	NR	[38]
19	83/F	Pfizer-BioNTech/1°	6	AchR+	NR	Generalized	P/IV-IG/S	NR	NR	[38]
20	77/M	O.A ChAdOx1/1°	3	AchR+	NR	Generalized	P/PLEX/S	NR	NR	[38]
21	53/M	O.A ChAdOx1/1°	1	AchR+	diplopia	Generalized	P360 mg/S15 mg	Improvement at 1MTH	no	[48]
22	68/M	Sinopharm/2°	3	AchR+	Dysarthria/dysphagia	Oculobulbar	P180 mg/IVIG/S	Improvement	no	[49]
23	46/F	Pfizer-BioNTech/1°	2	Triple-seronegative	Monolateral ptosis	Generalized	P/PLAEX/S/M	Stabilization	no	[50]
24	46/F	Pfizer-BioNTech/1°	5	AchR+	LL weakness	Generalized	P/PLAEX/S/M	Stabilization	TH	[51]
25	64/F	Pfizer-BioNTech/2°	12	AchR+	NR	NR	NR	NR	NR	[30]
26 *	73/M	O.A ChAdOx1/1°	28	AchR+	Diplopia	Generalized	P360 mg/S12.5 mg/AZA	Improvement	T	

Patient no. 6 had a recent diagnosis of cutaneous psoriasis; Patient no. 7 reported a 6-month history of fatigue before vaccine administration; Patient no. 13 developed several autoimmune diseases: a flare of ADEM, de novo MG, and thyroiditis. Abbreviations: Abs: antibodies; AchR: acetylcholine receptor; ANA: antinuclear antibodies; AZA: azathioprine; F: female; GW: generalized weakness; IG: immunoglobulins; IV: intravenous; LL: lower limb; M: male; MTH: month; NR: not reported; OA: Oxford AstraZeneca P: pyridostigmine; PLAEX: plasma exchange; RF: rheumatoid factor; S: steroid; T: thymoma; TH: thymic hyperplasia; UL: upper limb. * Our case.

## Data Availability

Datasets generated are available from the corresponding author on reasonable request.
